# THE FAITH CARE FAMILY PROJECT: A PILOT STUDY PROTOCOL

**DOI:** 10.1093/geroni/igad104.1086

**Published:** 2023-12-21

**Authors:** Ishan Williams, Noelle Fields, Ling Xu, Fayron Epps

**Affiliations:** University of Virginia, Charlottesville, Virginia, United States; The University of Texas at Arlington, Arlington, Texas, United States; The University of Texas at Arlington, Arlington, Texas, United States; Emory University, Atlanta, Georgia, United States

## Abstract

The use of lay providers/trained volunteers, to address ADRD caregiver needs may provide a scalable and culturally congruent approach for bridging gaps in ADRD care, particularly for African American families. Research examining the church as a platform for raising awareness about ADRD suggests that churches are well-suited to providing support and services to families affected by ADRD. This research protocol describes the development and testing of the Faith Care Family Project (FCF), a lay provider intervention for African American family caregivers in a church setting to address caregiver burden, coping skills, and social support. Guided by the revised sociocultural stress and coping model, an explanatory sequential mixed methods design will be utilized to test the efficacy of the FCF intervention with a sample of 18 church volunteer-family caregiver dyads. Specifically, the study addresses four aims: 1) How do church members and pastors perceive the key components of the FCF and what are their recommendations for implementation? 2) Do the church volunteers gain knowledge of ADRD after the 6-hour, FCF training? 3) Do family caregivers report more knowledge of dementia, less caregiver stress and improved well-being, more social support, and better coping skills after they participate in the FCF? and 4) What are the experiences of participants with the FCF? Data are collected at baseline, mid-intervention, post- intervention, 3 months-post intervention and through qualitative interviews. These evaluation activities will allow us to determine the applicability of the FCF as an effective, lay provider intervention for African American ADRD family caregivers.

